# Honokiol bis-dichloroacetate (Honokiol DCA) demonstrates activity in vemurafenib-resistant melanoma *in vivo*

**DOI:** 10.18632/oncotarget.7289

**Published:** 2016-02-09

**Authors:** Michael Y. Bonner, Isabella Karlsson, Monica Rodolfo, Rebecca S. Arnold, Elisabetta Vergani, Jack L. Arbiser

**Affiliations:** ^1^ Department of Dermatology, Emory School of Medicine, and Winship Cancer Institute, Atlanta, GA, USA; ^2^ Department of Dermatology, Veterans Affairs Medical Center, Decatur, GA, USA; ^3^ Department of Experimental Oncology and Molecular Medicine, Fondazione IRCCS Istituto Nazionale dei Tumori via Venezian, Milan, Italy; ^4^ Department of Urology, Emory School of Medicine, Atlanta, GA, USA

**Keywords:** melanoma, vemurafenib-resistant, reactive oxygen, mitochondria, xenographs

## Abstract

The majority of human melanomas bears BRAF mutations and thus is treated with inhibitors of BRAF, such as vemurafenib. While patients with BRAF mutations often demonstrate an initial dramatic response to vemurafenib, relapse is extremely common. Thus, novel agents are needed for the treatment of these aggressive melanomas. Honokiol is a small molecule compound derived from *Magnolia grandiflora* that has activity against solid tumors and hematopoietic neoplasms. In order to increase the lipophilicity of honokiol, we have synthesized honokiol DCA, the dichloroacetate ester of honokiol. In addition, we synthesized a novel fluorinated honokiol analog, bis-trifluoromethyl-bis-(4-hydroxy-3-allylphenyl) methane (hexafluoro). Both compounds exhibited activity against A375 melanoma *in vivo*, but honokiol DCA was more active. Gene arrays comparing treated with vehicle control tumors demonstrated induction of the respiratory enzyme succinate dehydrogenase B (SDHB) by treatment, suggesting that our honokiol analogs induce respiration *in vivo*. We then examined its effect against a pair of melanomas, LM36 and LM36R, in which LM36R differs from LM36 in that LM36R has acquired vemurafenib resistance. Honokiol DCA demonstrated *in vivo* activity against LM36R (vemurafenib resistant) but not against parental LM36. Honokiol DCA and hexafluoro inhibited the phosphorylation of DRP1, thus stimulating a phenotype suggestive of respiration through mitochondrial normalization. Honokiol DCA may act in vemurafenib resistant melanomas to increase both respiration and reactive oxygen generation, leading to activity against aggressive melanoma *in vivo*.

## INTRODUCTION

The majority of human melanomas express mutations in BRAF [1], and because of this mutation, much effort has gone into targeting BRAF and downstream pathways [2, 3]. New targeted therapies have been developed to inhibit BRAF signaling and downstream pathways. These drugs often produce dramatic responses, but unfortunately, these responses usually last for a few months. Some melanomas with BRAF mutations demonstrate intrinsic resistance, in that there is very little response even initially to BRAF inhibition. [4] Several mechanisms of resistance have already been described, including NRAS, MAP2K1, NF1, and BRAF amplification [5–8]. In addition, splicing mutations in BRAF have also been described [4]. Combination therapy of BRAF and MEK has led to the development of the MEK2Q60P mutation [9, 10]. Reactivation of ERK signaling appears to be a common thread in many forms of BRAF and MEK activation. Finally, many melanomas do not express BRAF mutations but have high levels of ERK activation [11, 12]. Thus, there is an unmet need for additional therapies to overcome resistance to targeted therapies.

**Figure 1:**
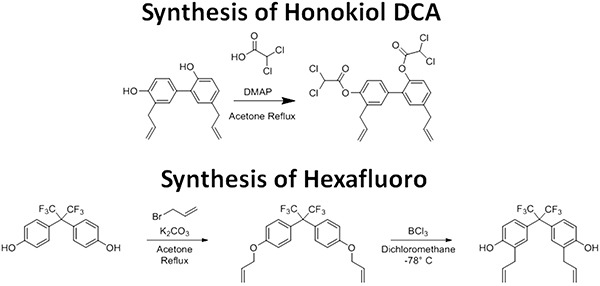
The synthetic scheme for Honokiol DCA and Hexafluoro

Honokiol is a small molecular weight compound derived from the tree *Magnolia grandiflora*. We were the first to demonstrate antitumor activity of honokiol *in vivo* [13]. Since then, we have found honokiol has been found to inhibit ras signaling and induce endoplasmic reticulum stress in tumor cells in multiple tumor types [14, 15]. Most recently, we have demonstrated that honokiol is an inducer of the mitochondrial gene Sirt3 [16]. In order to make honokiol more lipophilic and patentable, we synthesized a honokiol prodrug, honokiol bis-dichloroacetate (honokiol DCA) (Figure 1). In addition, we synthesized a novel honokiol derivative, bis-trifluoromethyl-bis-(4-hydroxy-3-allylphenyl)methane (hexafluoro) (Figure 1). Both honokiol DCA and hexafluoro demonstrated *in vivo* activity against A375 melanoma *in vivo*. In order to determine whether honokiol DCA had activity against vemurafenib resistant melanoma, we assessed the ability of honokiol DCA *in vivo* against LM36, a BRAF mutant melanoma and LM36R, a vemurafenib resistant clone of LM36 [17]. Honokiol DCA showed significant activity against the vemurafenib resistant LM36R but not against the parental LM36 cells. Unexpectedly, honokiol DCA induces Akt phosphorylation in the honokiol DCA sensitive A375 and LM36R cells, but not in the LM36 cells which are resistant to honokiol DCA. In order to determine the mechanism of this difference, we examined the ability of our compounds to induce superoxide in the tumor cell lines, since superoxide is a well known inducer of Akt phosphorylation [18]. LM36R cells, which are sensitive to honokiol DCA, lack expression of the major superoxide detoxifying gene manganese superoxide dismutase, while LM36, which is resistant to honokiol DCA, expresses high levels of MnSOD. Given that elevated Akt and superoxide are major players in advanced melanoma, honokiol analogs may be useful in treating these highly resistant subsets of melanoma.

## RESULTS

### Honokiol DCA and hexafluoro does not inhibit proliferation *in vitro*

We initially tested the antiproliferative activity of honokiol DCA and hexafluoro against A375 melanoma *in vitro* (Supplementary Figure S1). We found no significant difference in terms of *in vitro* proliferation. We have consistently observed that honokiol and derivatives tend to be more potent *in vivo* than *in vitro*, and *in vitro* inhibition of proliferation is not predictive of *in vivo* behavior with this family of compounds.

### Honokiol DCA and hexafluoro inhibits tumor growth in melanoma *in vivo*

**Figure 2:**
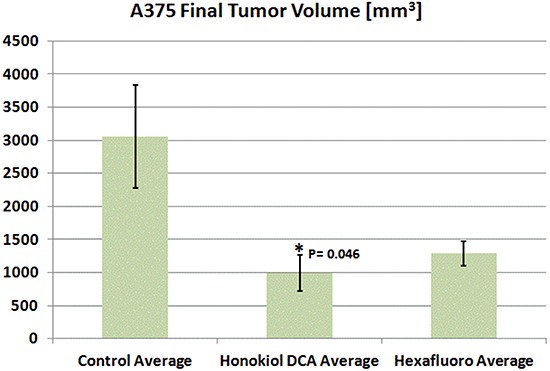
Honokiol DCA demonstrated significant antitumor activity *in vivo* against A375 malignant melanoma p <0.05. Animals treated with Hexafluoro showed marked but not significant tumor inhibition, *p* = 0.069. (*n* = 4).

We then assessed the *in vivo* activity of honokiol DCA and hexafluoro against A375 melanoma *in vivo*. A375 is a commonly used model of melanoma associated with mutant BRAF. Both honokiol DCA and hexafluoro demonstrated activity against A375 *in vivo*, but the activity of honokiol DCA, not hexafluoro, demonstrated significant tumor growth inhibition compared to the control group (Figure 2).

**Figure 3:**
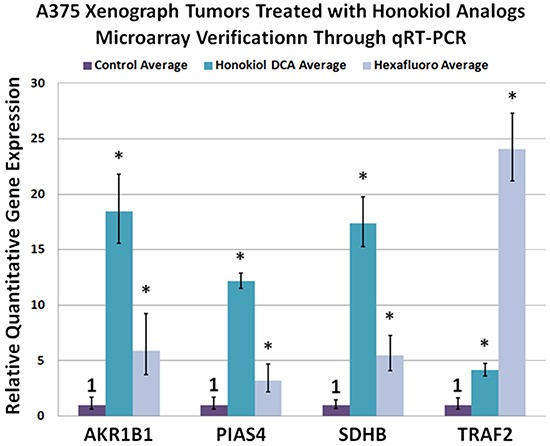
A375 xenograph tumors treated with honokiol analogs microarray verification through qRT-PCR Upregulation of SDHB (succinate dehydrogenase B), AKR1B10 (aldo ketoreductase 1B10), PIAS4 (E3 SUMO-protein ligase PIAS4 also known as protein inhibitor of activated STAT protein 4 (PIAS4) or protein inhibitor of activated STAT protein gamma) and TRAF2 (TNF receptor-associated factor 2) was observed via microarray analysis and further verified through qRT-PCR. (p < 0.05; *n* = 3)

In order to gain further insight into the mechanism of action of these small molecules, we harvested vehicle control and drug treated tumors and subjected them to gene array analysis (Supplementary Figure S3). A limited number of genes were commonly upregulated by both compounds in comparison to control and were confirmed by qRT-PCR (Figure 3). Honokiol DCA tended to show greater induction of genes than hexafluoro, and these genes may be useful as biomarkers for honokiol activity. Of interest, one of the genes that was commonly upregulated is succinate dehydrogenase B (SDHB), a tumor suppressor gene and respiratory enzyme [19, 20]. This is consistent with the known effect of honokiol induction of Sirt3.

**Figure 4:**
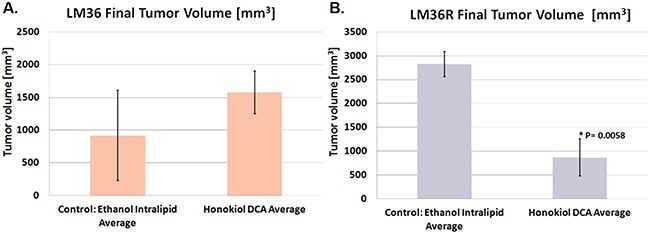
Honokiol DCA demonstrates significant antitumor activity *in vivo* against vemurafenib-resistant melanoma LM36R Honokiol DCA did not demonstrate significant antitumor activity *in vivo* against parental model LM36 **A.** despite having activity against the derived vemurafenib-resistant melanoma LM36R **B.** suggesting acquired signaling differences through resistance of BRAF inhibition. (p < 0.05; *n* = 4)

A major unmet need in treatment of human melanoma is drugs that have activity against vemurafenib resistant melanoma [21, 22]. We tested the ability of honokiol DCA to inhibit the growth *in vitro* (Supplementary Figure S2) as well as *in vivo* (Figure 4) of LM36 and LM36R, a pair of cell lines with BRAFV600E mutations that are sensitive and resistant to vemurafenib respectively [17]. LM36R, the more aggressive cell line, was inhibited *in vivo* by honokiol DCA significantly, while there was little effect on the sensitive and less aggressive LM36 parental cell line (Figure 4).

**Figure 5:**
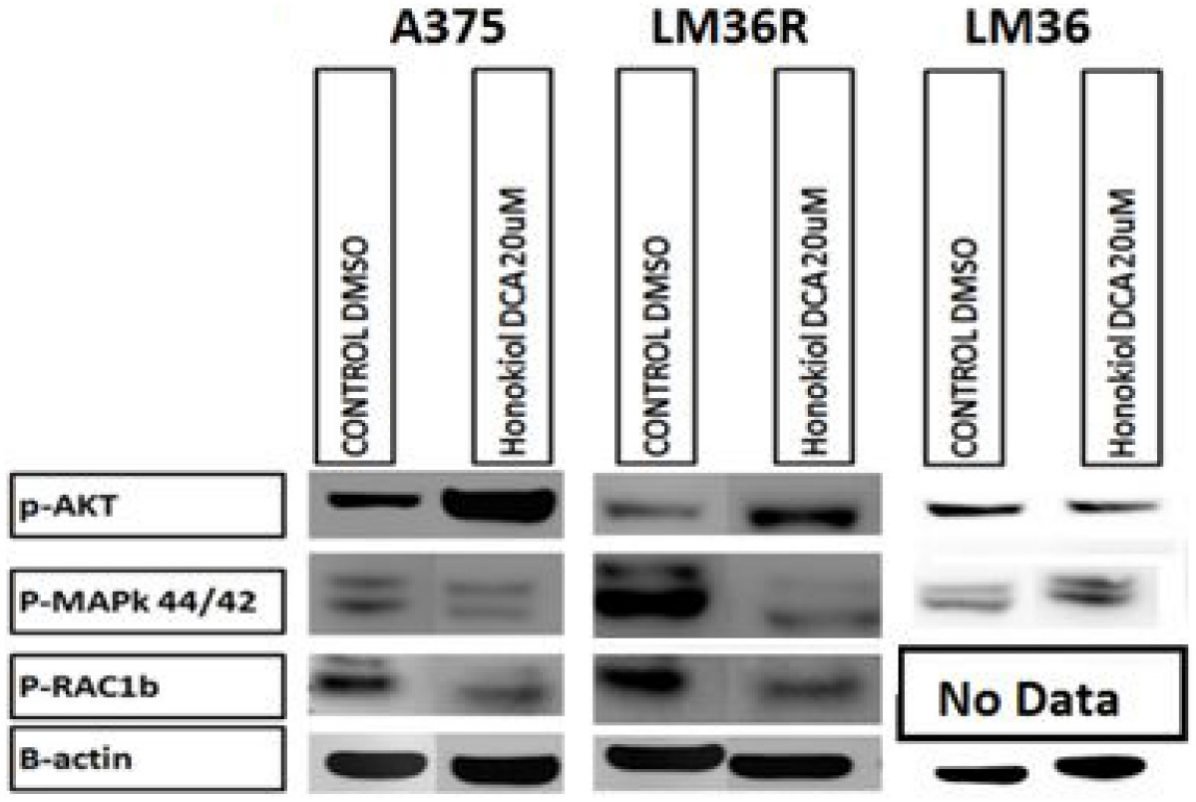
Western blot analysis show increased levels of p-AKT S473 on human melanoma cell lines A375 and vemurafenib resistant LM36R when treated for 24hrs at 20 μM AKT activation was unchanged in the parental melanoma cell line LM36. The effect on Honokiol DCA sensitive A375 and LM36R may be due to downregulation of p-42/44 MAPK, while LM36 is insensitive to Honokiol DCA. B-actin was used as a loading control. This experiment represents a single western blot analysis for p-Rac1B and triplicate experiments for p-AKT S473 and p-42/44 MAPK.

We assessed the signaling effects of honokiol derivatives against the melanoma cell lines, with particular attention to MAP kinase and Akt signaling, since these have been shown to be important mediators of melanoma growth (Figure 5). Surprisingly, honokiol DCA induced phosphorylation of Akt in the more aggressive but sensitive LM36R and A375 cells, but not in the less aggressive LM36 cells. The increase in Akt phosphorylation despite the effective antitumor activity suggested that ROS generated from the mitochondria might be activating Akt [23]. In addition, both phosphorylation of MAP kinase and levels of Rac1b were reduced.

Since Akt phosphorylation is often induced by reactive oxygen, we decided to assess whether reactive oxygen levels were affected by drug treatment. Two major sources of ROS in tumor cells are NADPH oxidases, which are rac dependent, and mitochondrial ROS, which is not rac dependent. Given that drug treatment decreased rac expression, if ROS is induced, the likely source would be mitochondrial rather than NADPH oxidase based.

### Honokiol DCA and hexafluoro induce elevated mitochondrial ROS production

**Figure 6:**
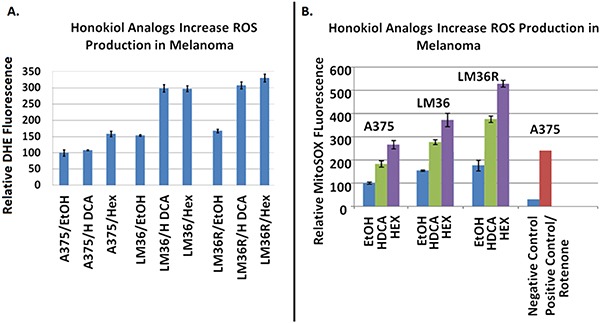
DHE fluorescence assays show Honokiol DCA and Hexafluoro increase overall superoxide levels **A.** Through the MitoSox Fluorescence assay Honokiol DCA and Hexafluoro demonstrate increased mitochondrial derived reactive oxygen production, indicating that they may act against melanoma through a reversion of the Warburg phenomenon. **B.** The combination of increased mitochondrial ROS plus NFkB inhibition may lead to selective tumor cell death.

**Figure 7:**
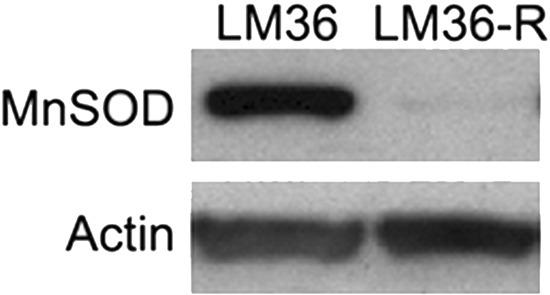
Western blot analysis comparing LM36 and LM36R MnSOD levels shows a loss of MnSOD expression in LM36R when compared to its parental cell line LM36 MnSOD expression levels may explain divergent sensitivity to Honokiol DCA between LM36 and LM36R. Actin was used as loading control.

**Figure 8:**
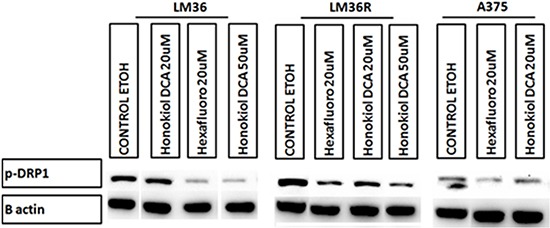
Western blot analysis show decrease levels of pDRP1 (S616) on human melanoma cell lines A375 and vemurafenib resistant LM36R when treated for 24hrs at 20μM The decrease in DRP1 phosphorylation suggests a possible mechanism for Honokiol DCA and Hexafluoro may be through inhibition of mitochondria fission.

Using DHE fluorescence, we assessed superoxide expression in response to treatment. Hexafluoro induced superoxide in all three cell lines, while honokiol DCA induced superoxide in 2 out of 3 cell lines (Figure 6A). We then examined mitochondrial derived superoxide using MitoSox fluorescence (Figure 6B). Both honokiol derivatives induced mitochondrial superoxide in all three cell lines. Induction of superoxide was more robust in the LM36R cells compared with LM36, so we examined expression of MnSOD in LM36 and LM36R. Expression of MnSOD was nearly absent in LM36R, while it was highly expressed in LM36 (Figure 7). Decreased phosphorylation of DRP1at S616 was also observed with treatment of both honokiol DCA and Hexafluoro (Figure 8). Given that phosphorylation of DRP1 at S616 is required for mitochondrial fission, the decreased phosphorylation of this site indicates a transition to mitochondrial fusion. While all three cell lines have elevated levels of phosphorylated DRP1, the honokiol DCA sensitive LM36R and A375 appeared to have a greater decrease in phosphorylation than the honokiol DCA resistant (and less aggressive) LM36 (Figure 8). The combination of induction of mitochondrial fusion in the absence of MnSOD may cause selective inhibitory effects on aggressive tumors lacking MnSOD.

## DISCUSSION

Melanoma is a common tumor that is well known for early metastasis and resistance to chemotherapy, antiangiogenic therapy [24–26], and radiation [27]. Recently, genetic subsets of melanoma have been characterized, especially with known driver mutations such as BRAF, NRAS, GNAQ, RAC1, C-KIT, and others [28]. Based upon the new knowledge of genetic alteration in melanoma, therapeutic interventions have been designed to treat melanoma. Most prominent among these are BRAF and MEK inhibitors. While BRAF mutant melanomas often respond to BRAF/MEK inhibition, in most cases the response is short lived. A major mechanism of resistance is activation of alternative signaling pathways [8].

One of the major signaling pathways that is not addressed by current therapies is superoxide. In what has been called the reactive oxygen driven tumor, superoxide can inactivate multiple tumor suppressors, such as p53, PTEN, IkB, and protein phosphatases [29]. Superoxide is a double edged sword for tumor cells, as it can serve as a tumor signaling pathway, or it can be used to kill the tumor cell [30]. Honokiol is a natural product that has attracted much attention because of its broad antitumor activity [31], and has been described as having pro-oxidant or anti-oxidant properties [32–34]. The precise context of honokiol's activity as an anti-oxidant or pro-oxidant is not fully understood. Our group was the first to demonstrate *in vivo* activity against established tumors [13]. Since this finding, we and others have demonstrated antitumor activity against epithelial, hematopoietic and sarcoma types of malignancy [13, 35, 36]. Honokiol has been demonstrated to have anti-invasive properties, anti-metastatic properties, and chemopreventive properties. Most recently, honokiol has been found to be a potent activator of the mitochondrial deacetylase Sirt3 [16], which is associated with an increased respiratory phenotype and fused mitochondria. Mitofusin2, associated with mitochondrial fusion, is also an antagonist of ras activation [37, 38], and induction of mitochondrial fusion could thus potentially explain the ras antagonism seen with honokiol [15]. Finally, the full potential of ras and raf oncogenes to transform is associated with defective fission type of mitochondria [39].

Delivery of natural products is a major hurdle in their translation to the clinic. Two major barriers to translation of natural products are multiple mechanisms of activity and the difficulty on obtaining intellectual property production on natural products with known structure. We believe that the ortho-allyphenol moiety is crucial to the activity of honokiol, based upon prior structure-function studies of the native honokiol molecule [13]. In order to test this, we have synthesized a novel honokiol analog with 2 ortho-allylphenol moieties containing a single carbon spacer (hexafluoro). This molecule is synthesized in 2 steps from industrially available precursors. In addition, we modified the natural product honokiol into a prodrug through esterification, which maintains activity *in vivo*.

Despite lack of efficacy in *in vitro* proliferation assays, both honokiol DCA and hexafluoro were both active *in vivo* against A375 melanoma, a BRAF mutant melanoma xenograft model. The NCI 60 proliferation inhibition index is commonly used as a screening index for candidate drugs [40]. Intriguingly, the antitumor properties of our analogs would not have been discovered based upon antiproliferative studies, as our compounds are not highly active as antiproliferative agents in culture. The energy requirements for proliferating in tissue culture may be very different from those required to create a 3 dimensional tumor under hypoxic conditions, and given the effects of our compounds on mitochondrial metabolism, *in vivo* conditions were required to demonstrate the effect of our compounds. Gene array analysis revealed few genes commonly upregulated by both compounds, including succinate dehydrogenase b (SDHB), which is involved in respiration and is a known tumor suppressor gene. Given that we have recently found that honokiol is an activator of Sirt3, this data suggests that honokiol derivatives are acting at least in part through induction of mitochondrial fusion, consistent with decreased phosphorylation of DRP-1 (S616) [41, 42].

Treatment of LM36R with honokiol derivatives leads to an increase in mitochondrial reactive oxygen species. One of the major mechanisms of detoxification of mitochondrially derived ROS is through manganese superoxide dismutase (MnSOD) [43]. A375, which is sensitive to honokiol derivatives, has been demonstrated to have extremely low levels of MnSOD, and is moderately resistant to vemurafenib [44, 45]. We compared expression of MnSOD in less aggressive LM36 and more aggressive LM36R and found that expression of MnSOD is nearly absent in LM36R compared with LM36 (Figure 7). Tumors that have low levels of MnSOD are known to be highly aggressive and absence of MnSOD may be a novel mechanism of resistance to vemurafenib. In addition, the absence of MnSOD may be a biomarker of sensitivity to honokiol derivatives. The resistance of the less aggressive LM36 to honokiol DCA is intriguing. The LM36 appear to have little response to 20 mM honokiol DCA in terms of phosphorylation of DRP1 compared to the more aggressive LM36R and A375 cells. The combination of lack of effect on DRP1 phosphorylation and thus mitochondrial fusion may allow the less aggressive cells to survive reactive oxygen stressors better than tumor cells which both lack the detoxification enzyme MnSOD and have increased mitochondrial fusion. Finally, honokiol derivatives may prove to be useful in killing cells with defective detoxification of mitochondrial ROS by increasing the biogenesis of these mitochondria and thus selectively killing tumors with defective elimination of ROS. Thus the context of the tumor cell may determine the role of whether a drug is a pro or anti-oxidant. In tumor cells that contain defective mitochondria or detoxification systems, activation of mitochondrial fusion could potentially amplify mitochondrial induced reactive oxygen, while in cells with normal mitochondria, honokiol could stimulate an antioxidant activity by stimulating normal mitochondrial biogenesis [46]. Treatment with honokiol derivatives may selectively target tumor cells with defective mitochondria, which often are the most resistant to current therapies.

## MATERIALS AND METHODS

### Synthesis

### Honokiol bis-dichloroacetate (Honokiol DCA)

Honokiol DCA was synthesized according to a procedure previously described by us [47]. Briefly, honokiol (1.0 g, 3.7 mmol) was dissolved in dry dichloromethane (200 mL), followed by addition of 4-dimethylaminopyridine (200 mg) from Sigma-Aldrich (St. Louis, MO). The reaction was heated to 40° C while stirring and dichloroacetylchloride (1.45 mL, 15 mmol) was added dropwise over 10 min. Next, the reaction mixture was refluxed for 5 h, after which all starting material was consumed according to TLC. After cooling the solution was washed with brine, dried over Na2SO4, filtered and concentrated under reduced pressure. The crude product was purified by column chromatography on silica gel (ethyl acetate/hexanes 1:9), which resulted in the wanted product (1.6 g, 76%). The obtained NMR was in accordance with our previous publication [47].

NMR spectra were recorded in deuterated chloroform (CDCl3) with a Varian INOVA 400 MHz instrument, calibrated using residual undeuterated chloroform (1H: δ = 7.24 ppm) as internal standard. The following abbreviations, or a combination thereof, are used to explain the multiplicities: s = singlet, d = doublet, t = triplet. High resolution mass spectrometry (HRMS) analysis was performed with a Thermo Scientific LTQ FT Ultra Hybrid mass spectrometer set on positive ionization.

### ortho-Allyl hexafluorobisphenol A (hexafluoro)

Hexafluorobisphenol A (1.0 g, 3.0 mmol) and anhydrous K2CO3 (2.07 g, 15 mmol) were dissolved in acetone (20 mL). The mixture was heated to reflux while stirring and allylbromide (1.0 mL, 11.5 mmol) was added dropwise. After 4h at reflux the solvent was removed in vacuum, and the remaining residue was taken up in ethyl acetate and washed with water and brine. The organic layer was dried over MgSO4, filtered, and concentrated in vacuum. The crude hexafluorobisallyloxyphenyl A (4,4′-(perfluoropropane-2,2-diyl)bis((allyloxy)benzene)) was used without further purification.

A procedure was adapted from the literature as follows: BCl3 (1M in CH2Cl2, 12 mL, 12 mmol) was added to a stirred solution of hexafluorobisallyloxyphenyl A (1.25 g, 3.0 mmol) in CH2Cl2 (10 mL) at −78°C under argon. The reaction mixture was allowed to reach room temperature followed by stirring under argon for 4h. The reaction was quenched by addition of H2O (20 mL), and the mixture was extracted with CHCl3 (3 × 20 mL). The organic layers were combined, washed with brine, dried over MgSO4, filtered, and concentrated under vacuum. The crude product was purified by column chromatography on silica gel (ethyl acetate/hexanes 1:9), which afforded hexafluoro as a yellow solid (1.15 g, 92%). 1H-NMR (CDCl3): δ 7.13 (d, J = 8.8 Hz, 2H), 7.08 (s, 2H), 6.77 (d, J = 8.8 Hz, 2H), 5.99 – 5.89 (m, 2H), 5.12 – 5.03 (m, 4H), 3.35 (d, J = 6.0 Hz, 4H). HRMS calculated for C21H19F6O2 417.12838, found 417.12877.

### Proliferation studies

Proliferation Studies were done using a Beckman Z1 Coulter cell counter. Cells were seeded 5 × 10^4^ cells per well in a 24-well plate. Cells were then treated the next day with 20 μM of honokiol, honokiol DCA, or hexafluoro from 10mM stock solutions in DMSO. Cells were incubated at 37°C with 5% CO2. Each compound was treated in quadruplicate wells. After 24 hours, cells were washed twice with PBS and trypsined. Cells were then counted in an isotonic solution.

### MTT assays

MTT assays were used to evaluate the effect of 72h drug treatment on LM36/LM36R cell growth as described previously [17].

### DHE assays

A375, LM36 and LM36R cells were treated with control, honokiol DCA, hexafluoro or vehicle for 24 hours. Cells were washed with PBS, digested with 0.05 % trypsin/0.53 mM EDTA and pelleted at 600g for 5 min. Cells were resuspended in 10 μM dihydroethidium (DHE) and incubated while gently shaking in the dark for 10 min and place on ice in the dark. Analysis of DHE fluorescence was performed on a Becton Dickinson FACScan flow cytometer, 10,000 cells were counted and analyzed by FlowJo 7.6.4. Mean values of the DHE fluorescence intensity were compared. Error bars represent the standard error of the mean of triplicate data points.

### MitoSOX assay

A375, LM36 and LM36R cells were treated with control, honokiol DCA, hexafluoro or vehicle for 24 hours. Cells were washed with PBS followed by the addition of 5 μM MitoSOX in phenol red free RPMI 1640. Cells were incubated for 30 mins at 37 C, 5 % CO2. MitoSOX was removed and cells were digested with 0.05 % trypsin/0.53 mM EDTA and pelleted at 600g for 5 min. Cells were resuspended in HANKS and placed on ice. Analysis of DHE fluorescence was performed on a Becton Dickinson FACScan flow cytometer, 10,000 cells were counted and analyzed by FlowJo 7.6.4. Mean values of the DHE fluorescence intensity were compared. Error bars represent the standard error of the mean of triplicate data points.

### Western blot analysis

Cells were plated in T25 flasks and treated when 80% confluent for 24 hours with 20 μM of each compound (honokiol, honokiol DCA, and hexafluoro. Blots were probed with antibodies for p-akt, p-mapk 42/44, p-p38, p-DRP1, total akt, total ERK, FOXD3, MDMX, p-Rac1b, MnSOD or beta actin were added at a concentration of 1:1000 in 5% non-fat dry milk in TBS and allowed to shake overnight in the coldroom (4°C) [48]. The next day, the blots were probed with anti-rabbit or anti-mouse HRP linked antibodies 1:10,000 (Cell Signaling) 5% non-fat dry milk in TBS with anti-rabbit or anti-mouse HRP linked antibodies (Cell Signaling). Super-Substrate from Thermofisher was used to activate the HRP linked secondary antibodies for development. The membranes were then developed using a Bio-rad docking station and camera. The software used was Bio-Rad Image Lab version 4.0.

### Gene chip analysis

Tumors from animals treated with control vehicle, honokiol, honokiol DCA, and hexafluoro were harvested and snapped-frozen in liquid nitrogen until RNA Extraction. RNA extraction was performed according to the Qiagen RNeasy kit. RNA samples were then submitted to Emory University's Intergrated Genomics Core for RNA quality analysis and gene expression assay. Gene expression analysis was performed using an Illumina HumaHT-12 v3 Expression Bead Chip and Gene Expression Module of Illumina's GenomeStudio Software package (v2011.1, Illumina).

### qRT-PCR

Gene chip analysis results were confirmed via qRT-PCR using the Applied Biosystems 7500 FAST Real Time PCR System. Briefly, cDNA was generated from RNA extracts using SuperScript VILO cDNA Synthesis Kit (Invitrogen) and the Eppendorf Mastercycler gradient. Taqman primers for SDHB, AKR1B10, PIAS4, TRAF2, and S18 (endogenous control) were used with TaqMan Fast Universal PCR Mastermix (2X) (Applied Biosystems).

#### *In vivo* studies

A375, LM36, and LM36R cells were injected 1 × 10^6^ cells per mouse, in groups of four. The cells were allowed two days of incubation before treatment. Honokiol DCA, hexafluoro were injected 5 times per week at 140 mg/kg in groups of 4 mice per compound. The injection cocktail was made by dissolving 16 mg of compound into 100 μl of absolute ethanol. The ethanol-compound solutions were then added to 1 ml of 20% soy-fat Intralipid (Frensenius Kabi) and vortexed vigorously [13]. Mice were injected with 0.25cc per mouse via intraperitoneal injections. The animals’ weights and their tumors’ lengths and widths were measured weekly. Tumor volumes were calculated via the tumor volume formula: (L × W^2^) × 0.52, with the smallest dimension being assigned the width and squared [49].

### Statistical analysis

#### Genechip analysis

Probe level intensity values were extracted and quantile normalization was performed using the Gene Expression Module of Illumina's GenomeStudio Software package (v2011.1, Illumina). Principle Component Analysis (PCA) and unsupervised hierarchical clustering were used to assess the variation of the samples’ expression profiles. To derive a refined list of genes most affected by honokiol DCA and hexaxfluoro compared to Control, the Significance Analysis of Microarrays (SAM; http://statweb.stanford.edu/∼tibs/SAM/) was used.

#### qRT-PCR

The statistical analysis was performed in triplicate using Applied Biosystems Sequence Detection Software (SDS Version 1.3.1). Using the software, relative quantification (RQ) values were obtain using the cycle time (CT) values which were first normalized to an endogenous control (S18) and then to experimental control and evaluated; p < 0.05.

#### *In vivo* studies

The statistical analysis for tumor volumes (defined by (L × W^2^) × 0.52, with the smallest dimension being assigned the width and squared) in the animal studies was performed on groups of 4, using Microsoft Office Excel 2007. P-values were determined using a two-tailed, two-sample equal variance (homoscedastic) student t test; p < 0.05.

## SUPPLEMENTARY FIGURES


