# Supplementary material for: Honokiol bis-dichloroacetate (Honokiol DCA) demonstrates activity in vemurafenib-resistant melanoma in vivo
Source: Oncotarget. 2016 Feb 9;7(11):12857–68. doi: 10.18632/oncotarget.7289 (PMC4914326; doi:10.18632/oncotarget.7289)
Supplement: Supplementary file 1 [file oncotarget-07-12857-s001.pdf]

SUPPLEMENTARY FIGURES

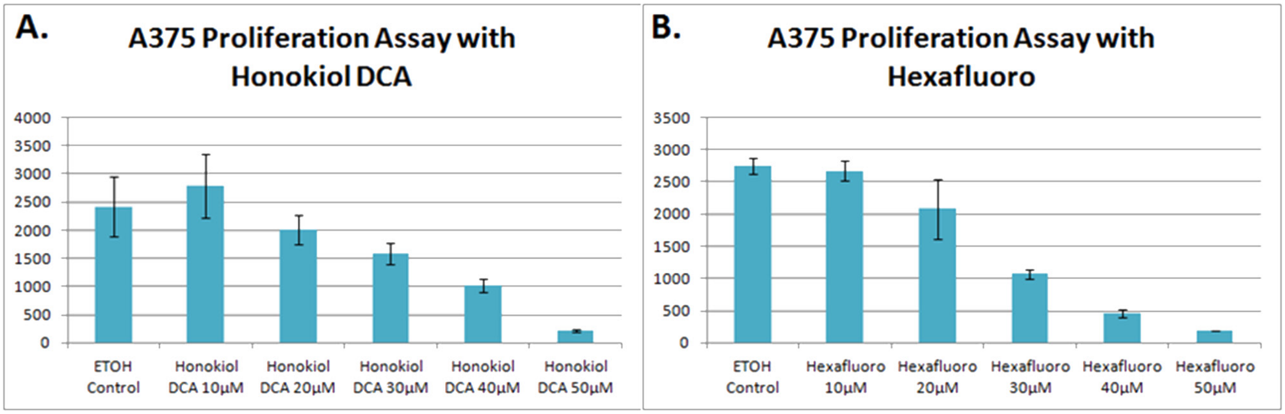

Supplementary Figure S1: Proliferation assay on A375 cells with Honokiol DCA, and Hexafluoro. Cells were treated for 24hrs in quadruplicate, * indicates $p < 0.05$ at 30 µM concentrations and greater.

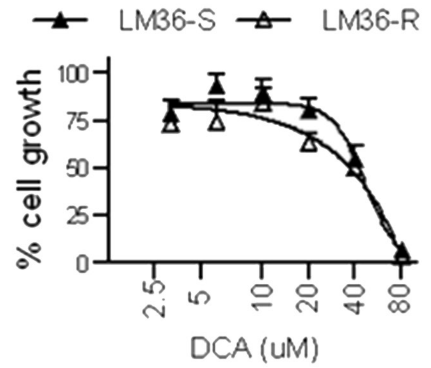

IC50 (μM)	LM36-S	LM36-R
DCA	49,3	not detectable
Honokiol	21,7	23,6
Hexafluoro	17	15,05

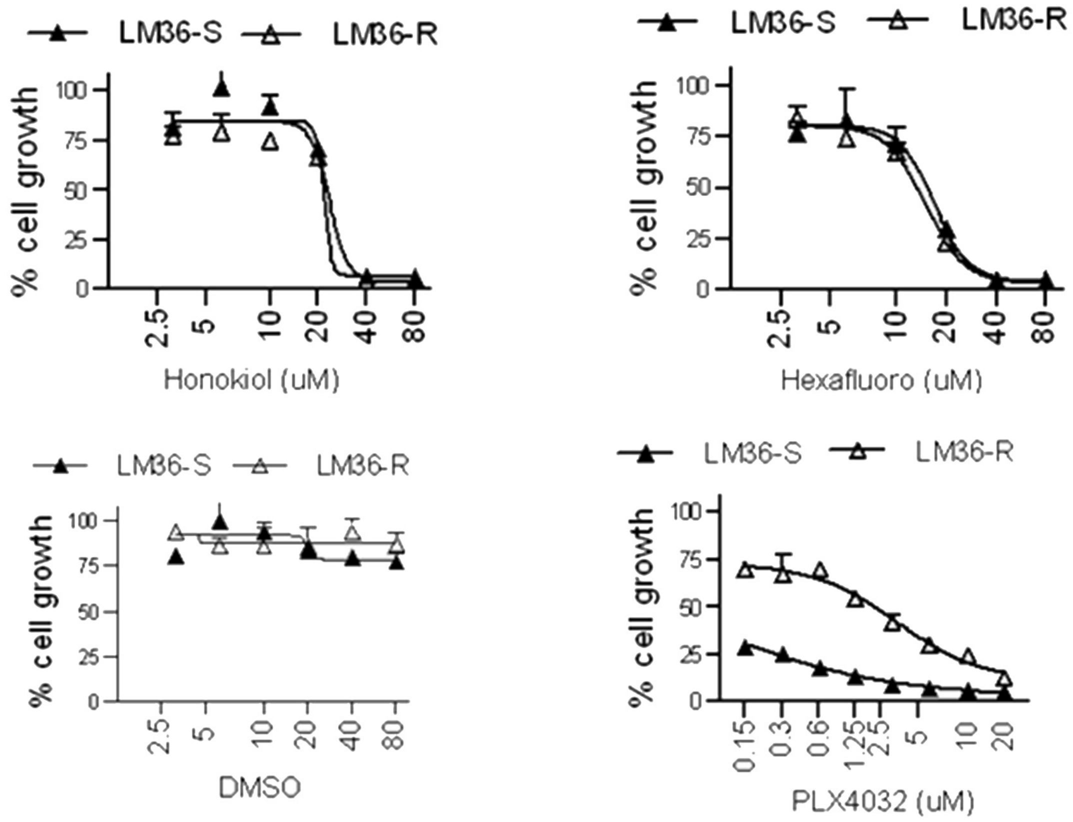

Supplementary Figure S2: Growth curves after 72 h treatment with Honokiol DCA, Honokiol, Hexafluoro and PLX4032 as detected by MTT assay in LM36 and LM36R cell lines. Calculated IC50 are indicated in the table. IC50 values were calculated by modeling results of growth inhibition assays using a nonlinear regression curve fit with a sigmoidal dose-response (variable slope).

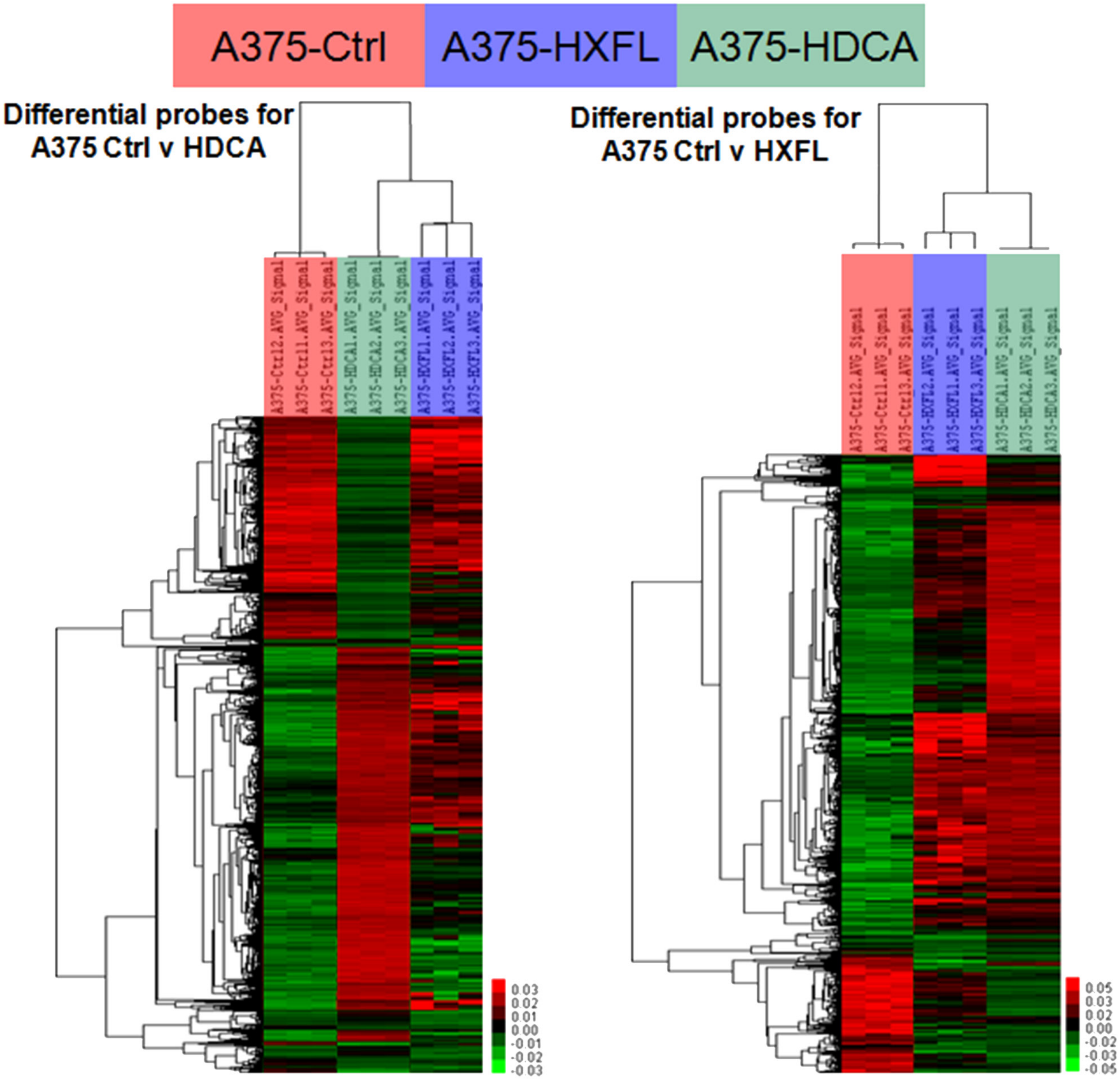

Supplementary Figure S3: Gene expression analysis heat map on A375 xenographs treated with Honokiol DCA and Hexafluoro an Illumina HumaHT-12 v3 Expression Bead Chip. Raw data is publicly available at Gene Expression Omnibus. GEO Acession number: GSE76956. http://www.ncbi.nlm.nih.gov/geo/query/acc.cgi?acc=GSE76956.
